# Generation of Pearl/Calcium Phosphate Composite Particles and Their Integration into Porous Chitosan Scaffolds for Bone Regeneration

**DOI:** 10.3390/jfb15030055

**Published:** 2024-02-21

**Authors:** Zhiyi Li, Ihtesham Ur Rehman, Rebecca Shepherd, Timothy E. L. Douglas

**Affiliations:** 1 School of Engineering, Lancaster University, Lancaster LA1 4YW, UK; z.li44@lancaster.ac.uk; 2 School of Medicine and Dentistry, University of Central Lancashire, Preston PR1 2HE, UK; iurehman@uclan.ac.uk; 3 School of Anatomy, Faculty of Health and Life Sciences, University of Bristol, Bristol BS8 1QU, UK; rebecca.shepherd@bristol.ac.uk

**Keywords:** pearl powder, calcium phosphate, chitosan, bone regeneration, porous scaffold

## Abstract

Bone tissue engineering using osteoconductive scaffolds holds promise for regeneration, with pearl powder gaining interest for its bioactive qualities. This study used freeze drying to create chitosan (CS) scaffolds with pearl/calcium phosphate (p/CaP) powders, mimicking bone tissue structurally and compositionally. Characterization included scanning electron microscopy (SEM) and mechanical testing. X-ray diffraction (XRD) Fourier-transform infrared–photoacoustic photo-acoustic sampling (FTIR−PAS), and FTIR- attenuated total reflectance (FTIR-ATR) were used to characterize p/CaP. In vitro tests covered degradation, cell activity, and SEM analysis. The scaffolds showed notable compressive strength and modulus enhancements with increasing p/CaP content. Porosity, ranging from 60% to 90%, decreased significantly at higher pearl/CaP ratios. Optimal cell proliferation and differentiation were observed with scaffolds containing up to 30 wt.% p/CaP, with 30 wt.% pearl powder and 30 wt.% p/CaP yielding the best results. In conclusion, pearl/calcium phosphate chitosan (p/CaP_CS) composite scaffolds emerged as promising biomaterials for bone tissue engineering, combining structural mimicry and favourable biological responses.

## 1. Introduction

Bone is an intricate and highly specialized variant of connective tissue which provides a sturdy framework that supports the human body and maintains its shape, while also providing attachment points for muscles and tendons [1,2].

Bone disorders can occur through physical damage, or as part of the ageing process. Treatments for bone defects include autografts, allografts, and xenografts [3]. However, the limitations of these methods include their limited availability, an increased risk of causing additional pain, nerve injury, cosmetic defects, infection, immune rejection response, and secondary fractures [4,5]. In addition, with an increasing aging population worldwide, more people are suffering from the chronic degeneration of bone tissues, which is also associated with disorders of other connective tissues, cartilage, and tendon [6,7]. These limitations have led to bone tissue engineering [4]. One strategy in bone tissue engineering is the biomimicry of natural materials, with the aim of controlling the synthesis of inorganic solids with an organic matrix. Carbonate minerals, which are components of sediments and sedimentary rocks, also play a role. These include calcium carbonate (CaCO_3_), one of the most abundant minerals on earth. CaCO_3_ potentially has important implications in biomineralization despite its relatively poor mechanical strength [8]. Over the history of the Earth, the primary source of marine carbonate minerals has shifted from abiotic precipitation to biogenic sources, and the simplest and most representative biominerals are pearls and shells [9].

The shell of bivalve molluscs, such as pearl oyster and freshwater pearl mussels, is a complex composite of calcium carbonate and organic (polymer) materials, essentially comprising two layers: an outer layer of prismatic calcite that often resembles chalk and an inner, pearly layer of nacre [10]. This inner layer is predominantly composed of aragonite, but frequently contains mixed crystals of calcite. Nacre, also known as mother of pearl, is a composite material produced by some species of mollusc that forms the inner structure of shells and pearls. This substance possesses a remarkable structure that is exceptionally tough [11].

Nacreous pearls are thought to form when a microscopic ‘irritant’ enters the mollusc shell and causes an increase in the production of nacre. The mantle edge of the mollusc shell secretes the organic framework and controls several aspects of the formation of the calcium carbonate crystals: their nucleation, growth, polymorphic structure, and even the positioning and elongation of crystal polygons within the concentric layers and in the surface region, which defines pearl quality [10]. The ancient Chinese people did not only value pearls as gemstones; they managed to apply pearl powders as medicinal ingredients in traditional Chinese medicines for thousands of years [12]. Moreover, the in vitro study of nacre with human osteoblast cells in 1992 represented the first utilization of nacre and pearls in bioengineering as biomaterials [13]. Recently, a number of studies have focused on the osteogenesis of pearls in polymeric scaffolds and hydrogels [14,15,16]; their antioxidant, anti-inflammatory, and immune-modulating properties make the application of pearls in wound healing and bone tissue engineering attractive.

Hydroxyapatite (HA) has been widely studied as one of the most representative bioceramics since 1950s [17,18]. It has a bony apatite structure and has been widely used as an artificial bone substitute because of its many positive biological properties, including biocompatibility, bioactivity, osteoconduction, osteoinduction, and osteointegration [17,18]. However, the common commercial highly crystalized structure of HA has a lower biodegradation rate compared with some ion-substituted, amorphous CaP, or poorly crystalline HA at both the nanoscale and microscale because of its relatively low osteoblast adhesion and ion releasing rate [19]. Furthermore, the direct use of HA has presented drawbacks compared with the use of ion-substituted or modified HA-based composites in different studies [20,21]. As a biomineralization precursor of the bone apatite generation process, amorphous calcium phosphate (ACP) has attracted considerable attention from researchers for the design of bone regeneration materials. In previous research, thermal gravimetric analysis (TGA), X-ray diffraction (XRD), scanning electron microscopy (SEM), and vibrational spectroscopy, including Fourier transform infrared spectroscopy (FTIR) and Raman spectroscopy, were considered as valuable techniques in studying ACP particles [22,23,24].

The hypothesis of this study was that the incorporation of pearl into bone scaffolds would improve the mechanical properties and cytocompatibility of bone scaffolds. This hypothesis was tested by incorporating pearl and CaP via a novel precipitation method. Subsequently, the porous CS composite scaffolds were fabricated in order to improve the properties of scaffolds, including their mechanical strength and biological activity. Characterization of the physiochemical and osteogenic properties of particles and scaffolds was performed.

## 2. Materials and Methods

### 2.1. Materials

Fresh water pearl powder (particle diameter in the micrometre range) was donated by the gN Pearl Group (Jingrun Zhenzhu, Haikou, China). For composite particle preparation, the following reagents were used for pearl/CaP synthesis: calcium nitrate tetrahydrate (Ca(NO_3_)_2_·4H_2_O, Acros Organics, Antwerp, Belgium); di-ammonium hydrogen phosphate ((NH_4_)_2_HPO_4_, VWR-Prolabo Chemicals, Darmstadt, Germany); ammonia solution (NH_3_·H_2_O, 35%, Thermo Fisher Scientific, Loughborough, UK); and ethanol (EtOH, ≥99.8%, AnalaR NORMAPUR^®^, VWR-Prolabo Chemicals, Paris, France; 99.8%, ThermoScientific™, Loughborough, UK). Commercial non-sintered HA particles (Plasma Biotal, CAPTAL^®^, Buxton, UK) were used for comparison analysis. All solutions were dissolved and diluted in Milli-Q water (Milli-Q^®^ Direct Water Purification System, Merck Millipore, Darmstadt, Germany). The medium-molecular-weight chitosan powders (100 kDa to 300 kDa, Acros Organics, Antwerp, Germany) were purchased from the Merck KGaA Group. The Dulbecco’s Modified Eagle Medium (DMEM, Gibco^®^, ThermoFisher™, Loughborough, UK), penicillin/streptomycin (P/S, 10,000 U/mL, Gibco^®^, ThermoFisherTM, Loughborough, UK), foetal calf serum (FCS, Sigma-Aldrich^®^, Dorset, UK), and the phosphate buffer saline (PBS, Sigma-Aldrich^®^, Dorset, UK) tablets were purchased from the Merck KgaA Group.

### 2.2. Fabrication of Composite Particles

The p/CaP composite particles were prepared by a precipitation method. In brief, Ca(NO_3_)_2_·4H_2_O with pearl solution and (NH_4_)_2_HPO_4_ solution were prepared as the sources of Ca^2+^, pearls, and PO_4_^3^. NH_3_·H_2_O was added dropwise to adjust the pH of the reaction solution. The reactant solutions were prepared at a stoichiometric ratio of 0.1 M Ca^2+^ and 0.06 M PO_4_^3−^ ions (equal to 1.67). For this, 0.1 M Ca^2+^ solution was prepared by dissolving the respective amount of Ca(NO_3_)_2_·H_2_O plus pearl powders in 200 mL of Milli-Q water; meanwhile, 0.06 M NH_4_^+^ solution was prepared in 200 mL of Milli-Q water.

The pH of both solutions was adjusted to 10.5 with NH_3_·H_2_O prior to starting the reaction. Afterwards, the alkaline (NH_4_)_2_HPO_4_ (40 mL) solution was added to the Ca(NO_3_)_2_ plus pearl (200 mL) solution dropwise at 5 mL/min under constant stirring. After the addition of (NH_4_)_2_HPO_4_ solution, the pH was adjusted to 10 before adding another round of NH_4_^+^ reagent solution (5 rounds, 200 mL in total). The pH adjustment process was repeated every hour to ensure the pH of the reaction solution remained at 10 ± 0.3. The stirring was stopped after 4 h; the reaction solution was incubated in a silicone bath at 80 °C overnight to achieve an ageing effect. After the overnight ageing process, the solution was filtrated by using a vacuum filtration system with a 250 mm Ø filtration paper (Whatman™, Buckinghamshire, UK), a Büchner funnel, and a vacuum pump (Fisherbrand™, Leicestershire, UK).

During the filtration, HA precipitates were washed with Milli-Q water; this was repeated several (4–5) times until the final pH of the filtrated water reached a value below 8.0. Then, the neutralized precipitates were collected with the filtration paper inside a glass dish, dried at 80 °C for 48 h, and crushed and ground in a mortar to obtain the fine HA powders.

### 2.3. Fabrication of Scaffolds

The chitosan solution was prepared by dissolving medium-molecular-weight chitosan powders (100 kDa to 300 kDa, Acros Organics, Antwerp, Germany) in 0.2 M acetic acid and heating and stirring at 50 °C for 1 h, followed by cooling to room temperature for over 6 h. Then, the ceramic components were added gradually to the solution and stirred overnight. The slurries were then poured into transparent plastic Petri dishes (Ø = 75 mm) and degassed at room temperature for 2 h to remove small bubbles. The samples were frozen in the freeze dryer at a decreasing temperature, from 0 °C to −20 °C (the gradient was −4 °C/h), for 24 h and then sublimated under a pressure of 0.7 mbar for over 24 h. Afterwards, the obtained scaffolds were processed in a neutralization step that transferred the scaffolds from the original Petri dishes by pressing the edges into larger Petri dishes. The 3M ethanol/NaOH (EtOH/NaOH) sol. (90–95%) was applied for the neutralization process; all scaffolds were immersed in the alkaline sol. for another 12 h. Later, a series of wash and rinse steps were performed, including (1) 15 min in Milli-Q water, (2) 5 min in PBS sol., (3) 15 min in 80% EtOH sol., and (4) 15 min in 100 EtOH sol. Then, the scaffolds were left half-covered in the hood to dry for 8–15 h. The details of all scaffold sample groups are given in Table 1 below.

### 2.4. Characterization

#### 2.4.1. FTIR Analysis

FTIR-PAS was used to analyse powder samples. Powders were poured into metal sample holders (Ø = 10 mm, × 2.5 mm depth), and the sample surface was levelled with the top of the holder. Spectral data were acquired in the mid-infrared region (4000–400 cm^−1^) at 8 cm^−1^ resolution with 200 spectrograph apertures, leading to an accumulated 128 scans. 

Scaffold samples were cut into rectangular specimens (10 × 10 mm^2^, 6 mm in height) prior to analysis, and the spectra were collected in the mid-infrared region by using an aperture of 150 with a resolution of 16 cm^−1^, leading to a total of 64 scans in FTIR-ATR mode. Both FTIR-ATR and FTIR-PAS spectra were collected by using a Nicolet™ iS50 spectrophotometer (Thermo Fisher Scientific Inc, Madison, WI, USA) in conjunction with Thermo Nicolet OMNIC™ (Version 9.5.9, Thermo Fisher Scientific Inc, Madison, WI, USA) software.

#### 2.4.2. XRD Characterization

XRD measurements were performed using the SmartLab (Rigaku SmartLab^®^, Rigaku Co., Ltd., Tokyo, Japan) X-ray diffraction meter. The resulting spectra were analysed using PDXL PDXL: Integrated X-ray powder diffraction software(Version 2.8.4.0 Rigaku Co., Ltd., Tokyo, Japan).

#### 2.4.3. SEM Analysis

Microstructure and morphology analysis of pearl, HA, and p/CaP composite particles and bioactive scaffolds were performed using a JEOL JSM-7800F Scanning Electron Microscope (SEM) instrument (Field Emission SEM, JEOL Ltd., Tokyo, Japan). Prior to imaging, samples were mounted onto 12.5 aluminium stubs using double-sided carbon adhesive tabs (12 mm) and sputter-coated with gold powder (5 nm) once.

For particle samples, before imaging, powders were dried in an oven at 80 °C for 48 h and stuck to carbon tabs on aluminium. For scaffold samples, all specimens were dried in the oven at 40 °C for 48 h and then sectioned to permit analysis of cross-sections, top surfaces, and bottom surfaces. Then, on each stub, three different surface scaffolds from the same sample were mounted horizontally to improve the electrical conductivity and to facilitate detailed imaging.

#### 2.4.4. Porosity Analysis

To determine the porosity of the scaffold, the circular specimen of weight W was immersed in a graduated cylinder containing a known volume (V_0_) of ethanol. Care was taken to ensure that no air bubbles emerged from the scaffold, and the cylinder was left for 5 min. The total volume of ethanol and ethanol-saturated scaffold was then recorded as V_1_. The volume difference (V_1_ − V_0_) represented the volume of the scaffold skeleton, while the residual ethanol volume after removing the scaffold from the cylinder was recorded as V_2_. The quantity (V_0_ − V_2_) was determined as the void volume of the foam. The total volume of the scaffold was then calculated as

V = (V_1_ − V_0_) + (V_0_ − V_2_) = V_1_ − V_2_. (1)

The density of the scaffold (d) was expressed as d = W/(V_1_ − V_2_), and the porosity of the scaffold (ε) was obtained by performing the following calculation:

ε = (V_0_ − V_2_)/(V_1_ − V_2_) (2)

In addition to porosity analysis, the SEM images of scaffolds were also analysed using ImageJ software (Version 1.53K). The results were calculated automatically by the distribution calculator from the software to obtain an average distribution of porosity.

#### 2.4.5. Compressive Properties Analysis

Cylindrical scaffolds of 10 mm in diameter and 6 mm in height were compressed at a rate of 5 mm/min using a Universal Testing Machine (Instron^®^ 3345, Norwood, MA, USA) with Bluehill^®^ Universal Software (Version 4.06, Norwood, MA, USA) The Young’s modulus was calculated based on the linear portion of the stress–strain curve.

### 2.5. In Vitro Cytotoxicity and Compatibility

All neutralized samples were cut with a cork borer of diameter 12 mm after soaking in 1% P/S-PBS solution overnight for rehydration. All specimens were sterilized by immersion in 70% EtOH solution for 2 h and evaporation of the ethanol for 24 h in a Biological Safety Cabinet (Type-II) (BSC) followed by an extra 15 min of exposure to UV. MG-63 cells, which are commonly used in related studies as an important in vitro cell model, were seeded onto sterile porous p/CaP_CS scaffolds in 24-well plates at 20,000 cells per sample in 50 mL of fully supplemented media. The samples were incubated using standard protocols at 37 °C and 5% CO_2_ in a fully humidified incubator to allow cell adhesion to the substrate for 3 h before 1 mL of fully supplemented media was added to each well [25]. The samples were cultured for 15 days with regular media changes.

#### 2.5.1. Cell Morphology

To observe cell morphology, after 14 days of incubation, scaffolds cultured with cells for SEM analysis were fixed at 2% overnight and then dehydrated in a series of ethanol treatments (70%, 80%, 90%, 100%) at 10 min intervals. The dehydration process was finished using HMDS (hexamethyldisilane) chemicals for 3 min, followed by drying in a desiccator for approximately 25 min prior to observation using SEM.

#### 2.5.2. Cell Cytotoxicity and Proliferation Analysis

The AlamarBlue^®^ (AB) assay is a non-cytotoxic, indirect way of evaluating cell viability and proliferation, and was performed in this study with measurements taken on day 1, day 3, day 6, day 10, and day 14. At determined culture intervals, cultured samples were washed with 1 mL of PBS with penicillin–streptomycin (P/S). Then, samples were immersed into 1 mL of resazurin solution in the wells of a 24-well plate, which was covered with aluminium foil to prevent contact with light and incubated for 4 h. After 4 h, the reduced resazurin media (resorufin), which transformed from blue into pink tones, were taken in triplicate from each well as 200 µL aliquots and poured into the wells of a 96-well plate in a zigzag pattern to prevent fluorescent interference from adjacent wells. The fluorescence values of the aliquot solutions were measured in an Infinite^®^ 200 PRO microplate reader (TECAN^®^, Tecan Group Ltd., Männedorf, Switzerland) using excitation and emission light wavelengths of 530 and 590 nm, respectively.

#### 2.5.3. Statistical Analysis

All of the data were statistically analysed with GraphPad Prism 8.0.2 software (Boston, Massachusetts USA. Data were reported as mean ± standard deviation (SD). Statistical significance was set at *p* < 0.05 and tested with two-way ANOVA.

## 3. Results

### 3.1. Composite Particle Properties

**Figure 1:**
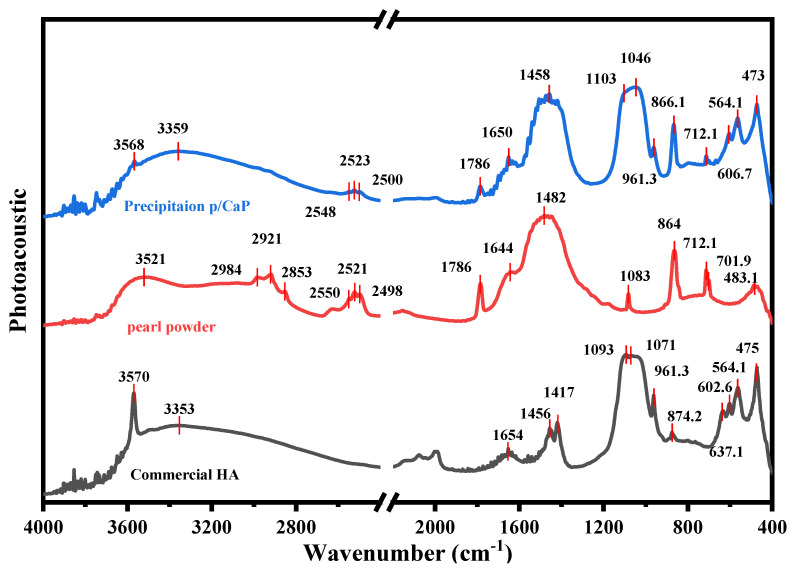
FTIR−PAS spectra of pearl (red), commercial HA (grey), and synthesized p/CaP (blue) composite powders obtained by precipitation method, along with the typical peak information. The spectra for the commercial HA powder does not demonstrate a peak in the ~1450–1490 suggesting that…… Visually, the spectra from pearl and synthesized p/CaP demonstrate a more similar structure when compared to commercial HA powder.

The FTIR−PAS spectra of the dried samples are presented in Figure 1. In pearl powders, the broad region at 3000–3400 cm^−1^ was attributed to the hydroxyl stretch, which was also characterized by broad H_2_O absorption. The peak at 2920 cm^−1^ was attributed to the ν(C-H) in the organic matrix of pearls [26]; the bands at 1430–1490 cm^−1^ were attributed to the bending vibration of CH_3_. The triple peak at 2520–2650 cm^−1^ was assigned to bicarbonate (HCO_3_^−^) O-H stretching.

Theoretically, carbonate ions have four vibrational modes, but three of them are more active under the IR spectrometer, including ν_1_ (1000–1100 cm^−1^), ν_2_ (840–910 cm^−1^), and ν_3_ (1350–1600 cm^−1^) [27]. In this study, the peak at 1787 cm^−1^ was attributed to the C=O groups in the carbonate ions, and the strong broad peak at 1656 cm^−1^ and the weak peak at 1414 cm^−1^ represented the in-plane asymmetric bending of CO_3_^2−^ (ν_3_). The peaks at 1082, 845, 712, and 701 cm^−1^ were attributed to two other CO_3_^2−^ vibrational modes. These identical IR peaks serve as distinctive markers for characterizing the samples as aragonite pearls [28].

Furthermore, the FTIR-PAS spectrum in Figure 1 provides further details of peak information in the region where the wave number is below 700 cm^−1^. As reported in a previous study [29], peaks at 630, 608, and 564 cm^−1^ were assigned to PO_4_^3−^ ν_4_ stretching, and the peak at 473 cm^−1^ was assigned to PO_4_^3−^ ν_2_ stretching.

**Figure 2:**
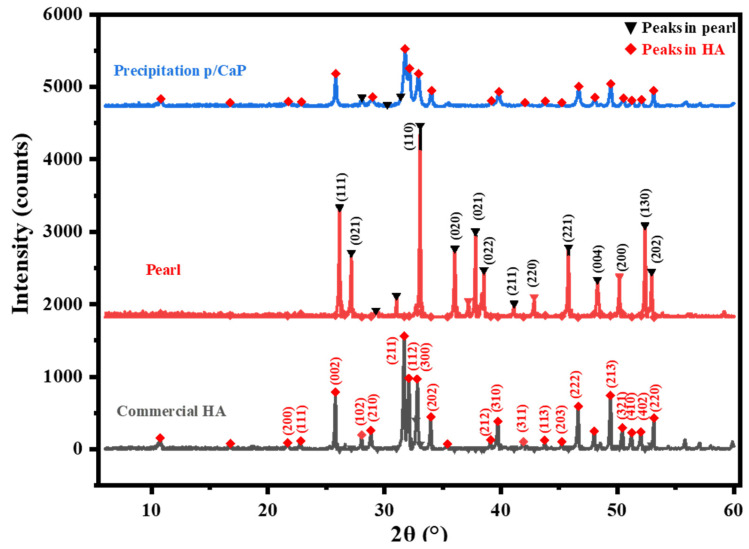
The comparison XRD patterns of synthesized p/CaP powders compared with the spectra of commercial HA and pearls. The diffraction pattern of the three composite samples: pearl (red), commercial HA (grey), and synthesized p/CaP (blue) composite powders.

In the diffraction pattern of the p/CaP samples, the main characteristic HA peak positions remained the same as in the synthesized HA samples, indicating that HA surrounded the surface of the pearl platelets, but the crystallinity of the obtained CaP appeared to be lower than the crystallinity of commercial HA, because the peaks were broader and less intense. Moreover, the sharp peak at the 2θ angle of 32.8° in the spectra of the sole HA shifted slightly to approximately 33.0° (±0.03°), which is closer to the characteristic CO_3_^2−^ peak at 33.05°. This peak shift indicates that the synthesized HA on pearl surfaces was connected by the chemical reaction, which is also in agreement with the FTIR-PAS results. As shown in Figure 2, the diffraction pattern of three composite samples demonstrated significant crystallinity differences; the CaP coatings adhered on the surfaces of pearl platelets synthesized by the precipitation method were more crystalline than samples obtained via other methods.

As Raynaud et al. reported in previous research, the XRD patterns of the unsintered or non-thermal treatment CaP composite was overlapped by the HA peaks; for instance, the CaO peak was stable at room temperature and could only be detected at 750 °C [30]. However, the single-phased CaP apatites could be created by the precipitation method, which, in this study, matched the more crystalized XRD pattern shown in the p/CaP composite particles.

**Figure 3:**
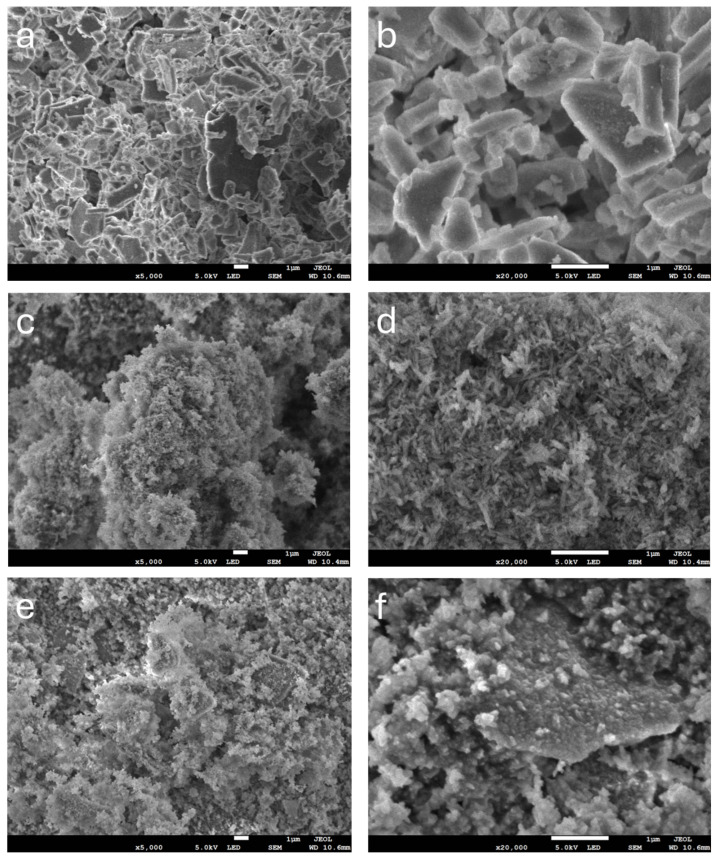
SEM microstructure images of synthesized HA samples and commercial HA samples at ×5000 and ×20,000 magnifications. (**a**,**b**) Pearl platelets used in this study; (**c**,**d**) synthesized HA particles for comparison; (**e**,**f**) p/CaP composite particles; newly formed amorphous CaP particles could be distinguished from the surface of pearl platelets. Scale bars at the bottom of images (**a**–**f**) are 1 μm.

The morphological analyses of the p/CaP composite samples are shown in Figure 3. As shown in the figures, embedded or attached crystals can be observed to be completely distributed over the pearl platelets. At lower magnifications (×5000), the synthesized amorphous CaP crystal agglomerates were easy to recognize. At higher magnifications, the morphology of composite particles displayed an amorphous interconnected structure. Compared with the synthesized HA samples at a magnification of ×20,000, the crystals attached to the pearl platelets displayed significant differences from those obtained by the sol–gel method and precipitation technology; however, they exhibited morphological similarities with the HA prepared by the hydrothermal method. The morphology of the surface of pearl powder and synthesized crystals clearly demonstrated that those crystals were formed at the nanoscale, with the entire surface appearing to be covered by the particles.

The SEM analyses of particle samples showed the diverse morphological properties of composite powders as a result of employing different synthesis methods. The pearl powder samples exhibited a significantly different morphology from the HA samples, that is, a typical platelet structure at the micro- and nanoscales. The commercial unsintered HA powders demonstrated typical rod-like pin structures. Samples obtained using the precipitation method showed a structure more similar to that of the commercial sample. Samples synthesized using the hydrothermal technique showed a more amorphous structural morphology, which differs from that found in previous studies, suggesting that the design of the synthesis process in this study, which incorporated an oven, requires further improvements to increase the crystallinity. Furthermore, the attachment of CaP crystals and pearl platelets, as demonstrated in the SEM images, indicates that the incorporation of pearl and CaP is feasible. A recent study conducted by Lotsari et al. demonstrated that the existence of amorphous CaP clusters is beneficial for promoting the growth of bone apatites [31].

### 3.2. Composite Scaffold Properties

**Figure 4:**
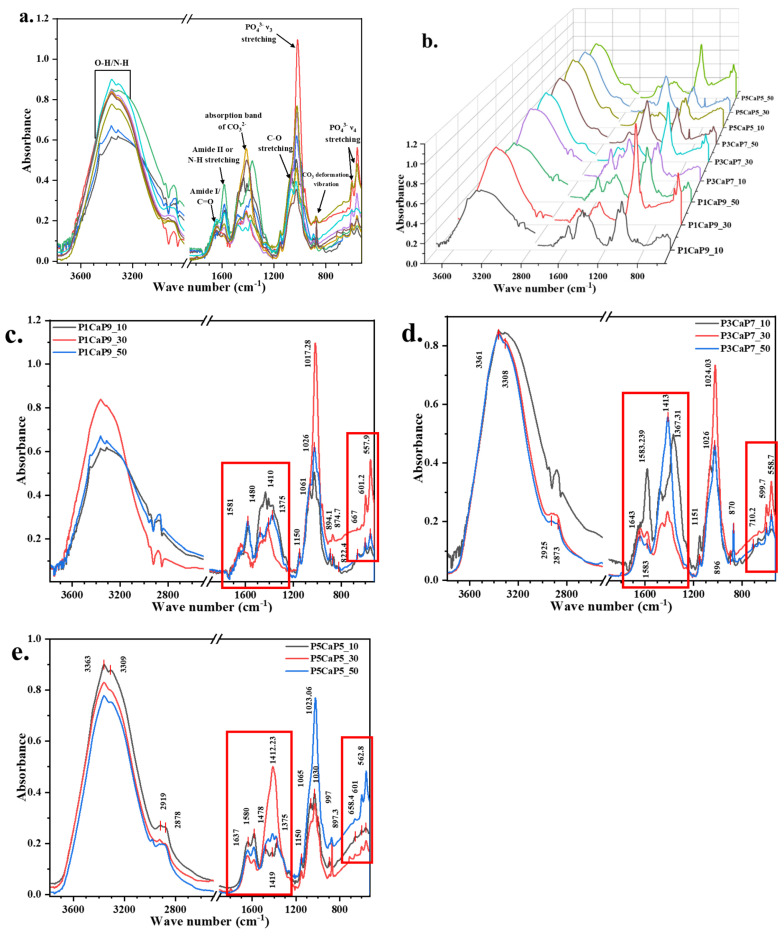
Comparison of FTIR−ATR patterns of three different groups of p/CaP chitosan scaffolds of different compositions. P_x_CaP_y_, x, and y stand for the pearl-to-CaP ratio; 10, 30, and 50 stand for the wt.% of composite particles in chitosan scaffolds. (**a**,**b**) The overlay and stack ATR spectra of 10, 30, and 50 wt.% P1CaP9_CS, P3CaP7_CS, and P5CaP5_CS groups; (**c**–**e**) ATR comparison spectra for composite p/CaP scaffolds in different composition groups. Red frames in subfigures (**c**–**e**) highlighted the main differences of P-O bands and C-O/Amide bands in different scaffold samples.

The ATR spectra of different scaffold samples, including 10, 30, and 50 wt.% pearl1_CaP9/CS (P1CaP9/CS); 10, 30, and 50 wt.% P3CaP7/CS; and 10, 30, and 50 wt.% P5CaP5/CS, are displayed in Figure 4.

**Figure 5:**
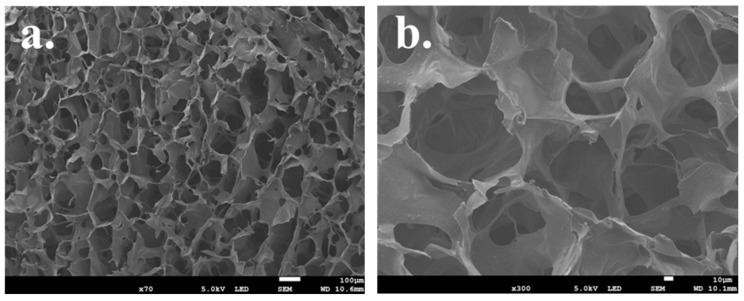

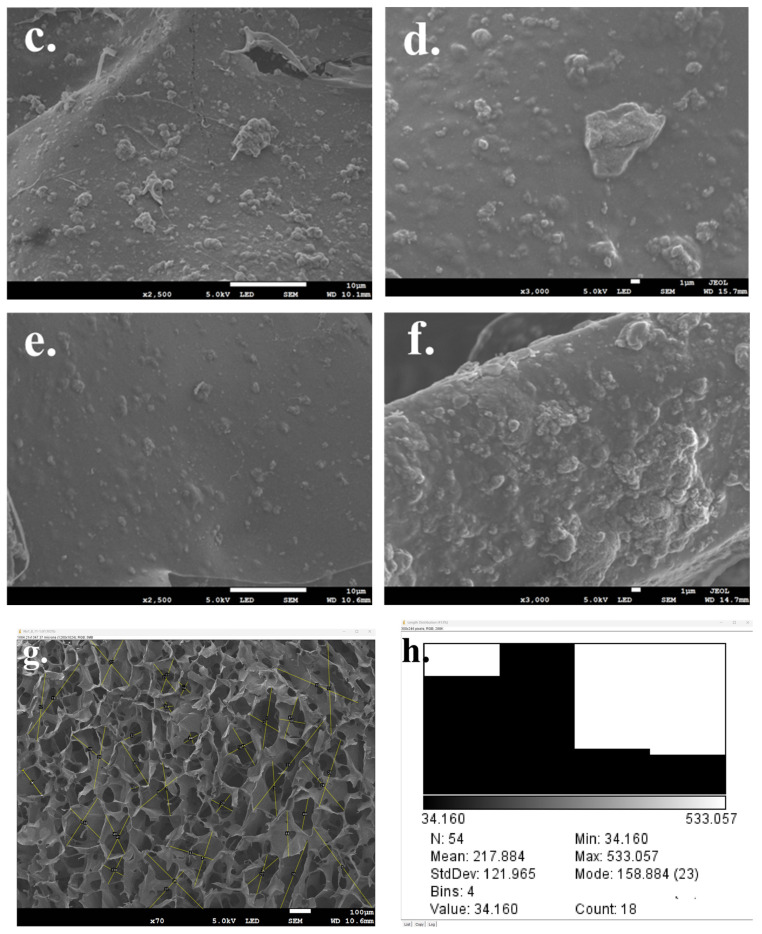
SEM microstructure images of pearl/CaP_CS scaffold samples under different magnifications. (**a**,**b**) Internal porous structure of the composite scaffolds (pore size ranging from 26 to 584 μm); (**c**–**f**) the morphology of p/CaP composite scaffolds with different pearl-to-CaP ratios and p/CaP compositions. (**c**,**d**) P3CaP7_30_CS scaffold, (**e**) P1CaP9_30_CS scaffold, and (**f**) P5CaP5_30_CS scaffold. (**g**,**h**) ImageJ was used for analysis of the diameter of pores in one SEM image for the composite scaffolds and the diameter distribution. (**a**) Magnification ×70, 100 μm; (**b**) magnification ×100, 10 μm; (**c**,**e**) magnification ×2500, 10 μm; (**d**,**f**) magnification ×3000, 1 μm.

The comparative spectrum for the p/CaP composite CS scaffolds is given in Figure 4. In comparison to the sole CS, pearl/CS, and HA/CS scaffolds, the p/CaP composite CS scaffolds exhibited the combined characteristic peaks of HA, pearl, and CS. Due to the chemical properties of pearl platelets, CaCO_3_ is dissolvable in acidic solution, and as can be seen in the morphological images displayed in Figure 5, the CaP particles were separately located on the surface of the pearl platelets. Therefore, this morphological structure does not allow HA to encapsulate the pearl platelet, resulting in the dissolution of CaCO_3_, which resulted in a different intensity of the CO_3_^2−^ deformation peak at roughly 869 cm^−1^. A peak shift was observed in the spectra in the region near 1020 cm^−1^, which was assigned to PO_4_^3−^ asymmetric stretching. With decreasing HA content, the P-O peak shifted from (P1CaP9/CS 30 wt.%) 1017 cm^−1^ to (P5CaP5/CS 50 wt.%) 1025 cm^−1^. The intensity of the PO_4_ band also decreased in agreement with the decreasing HA content. Moreover, the peak shift of the absorption carbonate band at around 1410 cm^−1^ towards 1360 cm^−1^ was observed in the spectra, and the intensity of the band increased proportionally to the increasing concentration of pearl powders. On the other hand, the crystallinity of the composite particles decreased, as evidenced by the decreasing intensity of the characteristic phosphate and carbonate peaks.

The comparative graphs presented in Figure 4c–e show the different weight percentages of scaffolds possessing the same pearl-to-CaP ratio. The comparative spectra were divided in accordance with the composition of the composite particles. The results show that when the weight percentages of mineral particles were increased, the intensity of the absorption carbonate peak at 1415 cm^−1^ increased, which covered the C-H bending peak and the C-N stretching peak existing in the chitosan matrix. This indicates that the dissolvement of peal powders released the free carbonated ions which were fixed by the cross-linked polymeric chitosan matrix. The phosphate ν_4_ bands in the region of 520–660 cm^−1^ were detected by infrared spectroscopy. Only peaks centred at 601 and 558 cm^−1^ showed well-defined sharp bands, which is in agreement with a previous study [29]. This result suggests that although some of the p/CaP composite particles can be dissolved by the HAc, a certain amount of CHA might also exist on the surface of the scaffold. In addition, it was noticed that in P1CaP9 and P3CaP7 composite CS scaffolds, 30 wt.% samples possessed slightly more intensity of the phosphate peak at around 1017–1025 cm^−1^, unlike in the p5h5 sample. This result might correspond with the detailed structural properties of p/CaP composites, thus requiring further investigation.

All scaffolds exhibited interconnected porosity, and it was possible to observe the distribution of ceramic crystals loaded onto the scaffold. As shown in Figure 5a–f, the combination of different concentrations of ceramic crystals and the different kinds of components did not lead to visible changes in the structure of scaffolds.

Similar pearl platelet morphology was observed for the pearl/CaP_chitosan composite scaffold in Figure 5c,d. Moreover, the amorphous CaP particles were found embedded in the wall of chitosan scaffolds and attached on the pearl platelets. With an increasing concentration of composite powders, p/CaP particles covered the walls of the chitosan scaffold more thoroughly and were dispersed on the surfaces more uniformly. Moreover, the increasing concentration of p/CaP particles also decreased the porosity of the scaffolds, which was confirmed by the porosity estimation analysis. The change in pearl-to-CaP ratios also affected the porosity of scaffolds; with a higher concentration of pearl platelets, the porosity of the scaffolds and the average pore size decreased slightly, but the structure was not affected, as demonstrated in Table 2.

This phenomenon may be associated with the agglomeration of p/CaP composite particles, which could block the pores of the CS matrix. The higher pearl-to-CaP ratio (from 10:90 to 50:50) additionally led to increased attachment of CaP onto the pearl surfaces, resulting in larger particle sizes and a subsequent decrease in the average pore size within the scaffold. This observation aligns with findings from previous studies [32,33,34].

The results of the porosity test were calculated and are summarized below in Table 3. Overall, the porosity of the scaffolds prepared in this research was relatively high, with all having a porosity over 60%, and the density of the scaffolds increased because of the incorporation of ceramic particles. In accordance with previous studies of porous scaffolds for bone regeneration [35,36,37], the porosity of scaffolds is an important parameter for the scaffolds but not the key factor. Higher porosity in scaffolds contributes to a greater surface area, favourable cell attachment, and protein adhesion on the scaffold in vivo [35,38]. On the other hand, restricted pore size and insufficient space for infiltration force cells to differentiate instead of proliferate; thus, relatively high porosity with an optimal pore size may be more appropriate for encouraging bone formation in vivo [39,40,41].

The density of all scaffolds was increased with the incorporation of ceramic particles. Compared with different compositions of pearl/CaP, with an increasing concentration of pearl powders, the density of scaffolds decreased, which might be related to the coating effect of CaP; the density of CaP is higher than that of pearl powders (3.16 g/cm^3^ > 2.73 g/cm^3^). The highest porosity among all scaffolds was 81.77%, which was achieved by the P1_CS scaffold, whilst the lowest porosity of scaffolds was 63.73%, which was observed for the P5CaP5_50 CS scaffold. Considering the diameter data from the SEM results, the porosity and the geometry of pores might be independent of these two factors and random. Statistically, the distribution of pore diameters shown in Table 2 is positively skewed, which means most of the pores generated by the freeze-drying technique have diameters in the range of 30 to 200 μm.

All scaffold samples exhibited an elastic–plastic–elastic behaviour, as shown in the curve, which might be due to their porous structure. A significant increase in compression strength and the elastic modulus were observed in comparison with the mean value between the sole CS scaffold and composite CS scaffolds. At the 70% strain point, the pure CS scaffold possessed the lowest mechanical strength, reaching approximately 74 kPa, while the pearl/CS 50 wt.% sample exhibited the highest strength at around 330 kPa.

The elastic modulus was calculated according to the stress–strain linear regression of the lower elastic region of all samples, and the R^2^ of all was ≥0.998. The elastic modulus of the composite CS scaffolds was within the region of articular cartilage tissues, which is reported to be in the range of 0.5 and 1.0 MPa [42,43]. But, when considering the Young’s modulus of human trabecular bone tissues, which was previously reported to be in the range from 0.9 GPa to 2.0 GPa, the test results reveal that the chitosan-based porous scaffolds might not be ideal compared with other hard tissue engineering scaffolds [44,45].

However, when more stress was applied to the scaffolds, due to their porous structure, the behaviour of the composite scaffolds showed a relatively good elastic performance (no significant break points were observed from the curves, except for the HA/CS scaffold samples) as well as compressive strength. This improvement might conceivably result from the increasing Ca^2+^ and carbonate groups from the pearl or pearl/CaP components interacting with the amino groups in the CS [46,47].

### 3.3. MG-63 Cell Growth on Scaffolds

**Figure 6:**
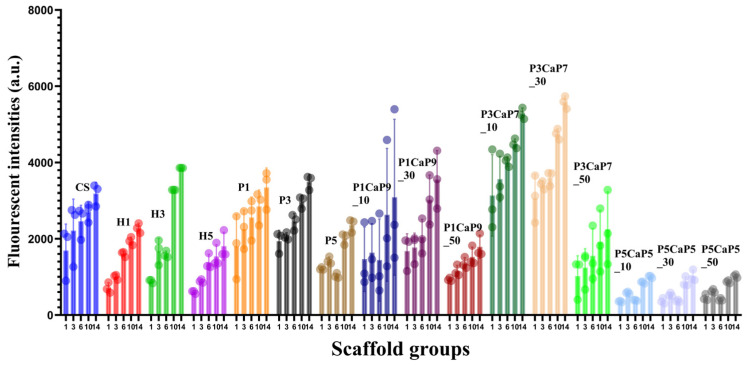
The cell proliferation and cytocompatibility analysed using the AB assay for up to 14 days of culture. Six groups of samples were tested, including the sole CS, pearl_CS group, HA_CS group, pearl/CaP(10:90)_CS group (P1CaP9), pearl/CaP(30:70)_CS group (P3CaP7), and pearl/CaP(50:50)_CS group (P5CaP5).

Following the measurement of particles and scaffold properties, the scaffolds underwent evaluation for the in vitro culture of MG-63 cells (Figure 6).

The fluorescent intensities of samples between different groups were in the vicinity of 500 to 3000 on day 1, and most samples showed a slight increase during the first week of culture. The different initial fluorescent intensities of different sample groups are probably due to the affinity between the cells and the scaffolds and the fact that the migration and attachment processes on different samples might be influenced by different factors, including the surface energy and porosity of scaffolds. Obviously, the P5CaP5 group showed the worst biocompatibility at each time point compared with the other p/CaP composite groups, the HA group, and the pearl group. This might be due to the concentration of CaCO_3_ content, with a high concentration of calcium carbonate being incorporated into the chitosan scaffold, as well as the lower porosity and the average pore size [48].

As time increased, the MG-63 cells on all scaffolds significantly proliferated. During the first week of incubation, all samples showed increasing proliferation of MG-63 cells, except the P5CaP5 group samples. Moreover, cells proliferated more significantly on composite scaffolds in the H1, H3, P3CaP7_10, and P3CaP7_30 groups than on the pure CS scaffold group, which indicates these groups’ good cytocompatibility and tendency to encourage proliferation of MG-63. From day 6 to day 14, compared with the first incubation interval, a slightly higher proliferation of cell metabolic activities was observed; the P5CaP5 group samples especially showed significantly increased fluorescent intensity on day 10. The highest cell viability was obtained for the P3CaP7_30 sample on day 14, which was similar to that of the P3CaP7_10 sample.

The visible cell proliferation and attachment morphologies on the scaffold samples were observed by SEM on day 14, with these findings supporting the results obtained from the AB assay.

**Figure 7:**
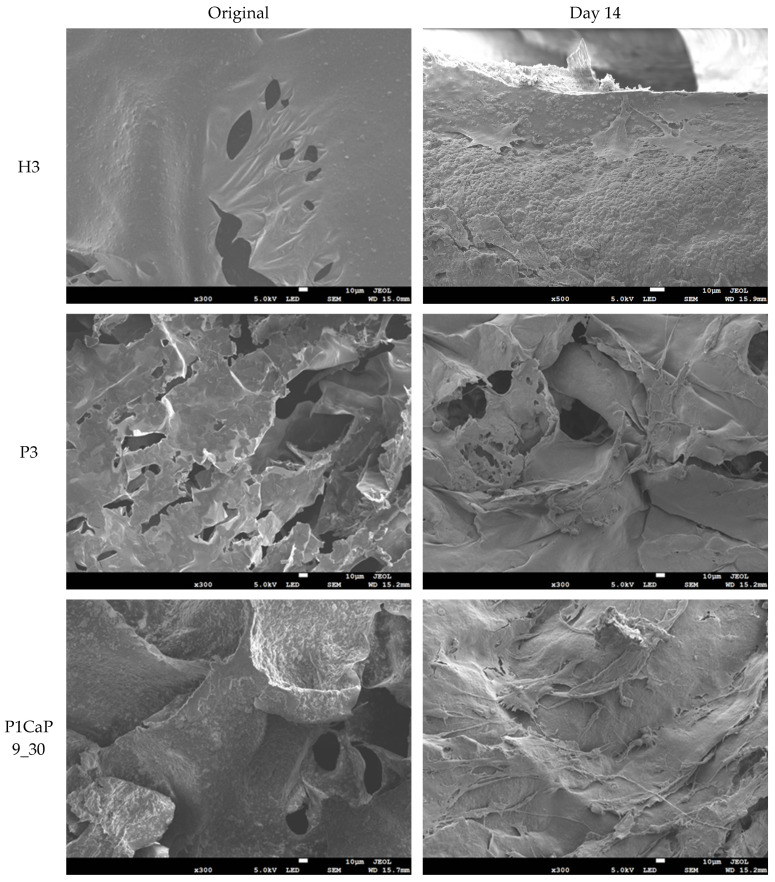

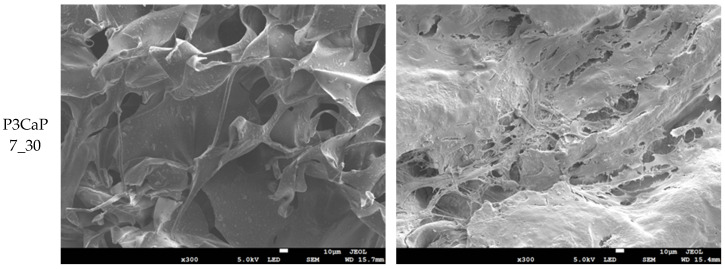
The comparative SEM images of various scaffolds, including the H3, P3, P1CaP9_30, and P3CaP7_30 scaffold samples and samples with grown MG-63 cells after 14 days of incubation. All images are at ×300 magnification, 10 μm scale bar.

As shown in Figure 7, all composite scaffolds exhibited a similar microstructure, with particles distributed on the chitosan matrix. On day 14, cells were observed to be successfully attached to the surface of each sample with extending morphology. Firstly, comparing the P3 and H3 samples, cells were successfully distributed on the top surface on both scaffolds, and obvious differences were seen between H3 and P3 in that more external cellular cross-linking morphologies could be observed in the P3 samples. It is revealing that pearl powder indeed affected the cell morphology on the scaffold surfaces. However, the cell numbers on the P3 scaffold were slightly reduced compared with those on the H3 scaffold, which corresponds with the result of the AB assay. In addition, the P1CaP9_30 and P3CaP7_30 scaffold samples showed a higher number of cell attachments compared with the P3 scaffold samples, and the cellular cross-linking structure could be more easily identified from the SEM images. Thus, the presence of the p/CaP composite particles not only led to cell proliferation but also promoted fibroblast migration in this research.

## 4. Discussion

The synthesis of composite powders with pearl and calcium phosphate (p/CaP) through three distinct methods, stemming from hydroxyapatite (HA) synthesis, represents an innovative approach. Amorphous CaP has garnered increasing attention in comparison with stoichiometric HA due to its crucial role in bone apatite nucleation and the mineralization process within the bone remodelling cycle. Previous studies, such as Raynaud’s [30], have suggested that amorphous calcium phosphate (ACP) may be sequestered and deposited via intracellular and vesicular mechanisms in the gap zones of the collagen matrix. This proposed role positions ACP as a transient phase, acting as a precursor for subsequent apatite growth. Complementary in vitro investigations, employing collagen as the organic scaffold, further substantiated the presence of ACP at the gap zones of collagen, serving as a precursor stage before transforming into apatite. Earlier studies by Boonrungsiman et al., Omelon et al., and Rustom et al. have provided additional insights into the growth mechanism associated with the transformation of amorphous CaP to apatite [49,50,51]. Consequently, the anticipated outcome for p/CaP composite particles is an enhancement in biocompatibility and osteogenesis properties.

The SEM analysis of p/CaP composite samples uncovered a compelling integration of embedded or attached crystals intricately distributed throughout the pearl platelets. Even at lower magnifications, the synthesized amorphous CaP crystal agglomerates were clearly visible, while at higher magnifications, an intricate, amorphous interconnected structure emerged. This unique amalgamation of pearl and CaP imparted distinctive morphological characteristics, particularly at higher magnifications, setting it apart from HA samples obtained through alternative synthesis methods. Importantly, the SEM images provide compelling evidence supporting the feasibility of incorporating both pearl and CaP in this study.

The attachment of CaP crystals and pearl platelets showcased promising interactions, substantiating the notion that amorphous CaP clusters, as observed in this study, play a pivotal role in fostering the growth of bone apatites. This observation aligns with the findings of Lotsari et al., underlining the critical importance of amorphous CaP clusters in the autocatalytic nucleation of HA and the formation of Ponsner’s clusters [31]. The SEM results offer valuable morphological insights, laying the foundation for further explorations into the potential applications of the pearl/CaP composite in the realm of bone regeneration and related fields.

The freeze-dried composite scaffolds in this investigation showcased interconnected and porous structures with homogeneity. SEM characterization of the porous morphologies across all scaffolds revealed dimensions ranging from 26 to 600 µm, aligning with the requisites for cell migration and proliferation in bone tissue engineering. The surface morphologies of the pearl, HA, and p/CaP composite scaffolds exhibited distinctive rough structures. The HA/CS scaffold displayed a pin-like morphology attributed to the unique crystal structure of commercial HA. Conversely, pearl/CS scaffolds showed platelet crystal distribution on scaffold walls. The p/CaP_CS scaffolds presented a morphology combining pearl platelets and HA, but SEM revealed more dispersed amorphous crystal agglomerations. 

FTIR-ATR spectroscopy was employed to characterize the chemical properties of the scaffolds. The limited differences in peak intensity between the bottom and top layers of all scaffold samples indicated a relatively homogeneous distribution of pearl, HA, and p/CaP composites, which is promising for their bioactive performance.

The mechanical properties of different scaffold groups demonstrated enhancements when comparing HA scaffold groups with the p/CaP scaffold groups. None of the scaffolds experienced breakage at 70% strain, and all specimens rebounded close to their initial state after testing. The highest mean compression strength at 70% in the composite chitosan scaffolds was noted at 330 kPa, specifically for the pearl 50 wt.% CS scaffold. Additionally, the highest elastic modulus reached was 0.87 MPa. 

This compressive test result is associated with the distinct morphological structures of pearl, HA, and p/CaP. As previously discussed, the platelet structure of pearl was more embedded in the chitosan matrix wall, while p/CaP scaffolds demonstrated larger agglomerations attached to the surface. Research by Zeng and Zhang [52] proposed that the incorporation of pearl can significantly enhance the mechanical properties of composite chitosan microspheres, with cationic compounds from chitosan particles actively interacting with the organic matrix of pearl shell powders [52]. This perspective provides insight into the enhancement of the mechanical strength of p/CaP chitosan scaffolds. However, increasing the pearl-to-CaP ratio to 50:50 resulted in larger particle sizes, causing clotting of internal pores, as indicated by the decreased porosity in the porosity estimation section, as well as changes in the elastic modulus.

In cell culture studies, scaffold compositions did not show any cytotoxicity effect on the cells, and cells gradually proliferated during the culturing for up to 14 days. In the experiment group, the cell seeding density of 20,000 cells per scaffolds (Ø: 12 mm × 5 mm) cultured in a 24-well plate by using 1 mL of growth media was found to be the optimal seeding condition. In this experiment, the initial cell attachment after 24 h was very different in samples, with the pearl chitosan groups demonstrating the worst cell attachment, while the composite p/CaP group had significantly better cell activity. After 14 days, the beneficial effect of pearl, HA, and CaP individually on increasing cell compatibility has been reported by different studies [15,53,54]. The effect of HA in contributing to the absorption of serum proteins including fibronectins and vitronectins, leading to the attachment of integrins and hence attachment of osteoblast precursor cells, has been reported [55].

In the present study, the cell viability of samples exhibited variations between test intervals, and the cell proliferation performance of all compositions showed distinct patterns. The p/CaP group scaffolds, particularly the P3CaP7_30 scaffold, demonstrated the highest terminal fluorescent intensity after the 14-day incubation period. This finding suggests robust cell proliferation and activity in this specific composition. However, it is noteworthy that the P5CaP5 group exhibited the lowest fluorescent intensity, indicating comparatively lower cell proliferation.

Relevant studies by Zhang and Zhang, Zhang et al., and Li et al. provide insights into the impact of varying concentrations of CaP and pearl on cellular activity and proliferation [14,54,56,57]. Zhang and Zhang focused on the uptake of macroporous chitosan/calcium phosphate composite scaffolds, revealing that increasing the concentration of CaP led to enhanced cellular activity and proliferation, supporting the formation of apatite [54]. Zhang et al. explored the incorporation of pearl powder content in PCL scaffolds, demonstrating that higher pearl contents, up to 80 wt.%, facilitated increased proliferation and osteogenesis [56]. Li et al. studied nano-pearl powder/chitosan–hyaluronic acid porous composite scaffolds and concluded that higher pearl concentrations (0 to 25 wt.%) promoted cell proliferation and osteogenesis [14].

Furthermore, the topography of various scaffolds has been shown to influence cell culture test outcomes in previous studies [58,59,60,61]. Serving as a significant physical cue, scaffold topography in this investigation was found to be closely associated with particle distribution and compositions. SEM images depicted distinct topographical variations among the four samples presented in Figure 7. Moreover, samples exhibiting greater numbers of grooves/pores and particles adhering to the scaffold surface displayed higher AB assay results, consistent with prior research findings. However, the precise mechanism underlying the observed enhancement in cell proliferation on these scaffolds remains elusive. Therefore, further investigations are warranted to comprehensively elucidate the intricate relationship between scaffold topography and composite particles. However, the poor cell proliferation reaction observed in the present study with a 50:50 ratio of pearl to CaP suggests a unique response that might be related to an unknown mechanism. This anomaly emphasizes the need for further in-depth investigation into the specific conditions and interactions at this particular composition ratio. Understanding the underlying factors influencing cell behaviour in response to different material compositions is crucial for optimizing scaffold design and enhancing their effectiveness in applications. The mechanism of precipitation attachment of pearl and CaP is not clear yet, and most of the work in this study only provides evidence of the properties of CaP. Moreover, as the SEM images suggest, amorphous CaP enveloped the surface of the pearl platelets, and the pearl platelets only provided extra Ca^2+^ and CO_3_^2−^, as shown in FTIR-PAS spectra. The organic matrix of pearls was not investigated in this study but will be considered in future experiments.

## 5. Conclusions

This study demonstrated the feasibility of preparing a p/CaP composite powder, the incorporation of p/CaP into chitosan scaffolds, and the non-cytotoxicity and the proliferation capability of a scaffold using MG-63 cell lines, as proved through in vitro biological testing.

The p/CaP composite powders were synthesized by the precipitation method. CaP covered the surface of pearl platelets. Pearl promoted the generation of CaP components during the synthesis process of HA, and the carbonate groups played an important role. 

FTIR−ATR analysis proved the distribution of p/CaP particles throughout the chitosan matrix quantitatively. Compressive mechanical analysis showed that the incorporation of p/CaP composite powder into the chitosan matrix enhanced the compressive strength.

P/CaP_CS scaffolds demonstrated a higher degradation ratio than the HA groups and a lower ratio than the pearl groups, indicating the existence of CO_3_ compounds, and amorphous CaP composite influenced the degradation process of the scaffold.

The fabricated porous p/CaP scaffolds were non-cytotoxic in the presence of MG-63 osteoblast cells. The 14-day cell culture results highlight the optimal performance of scaffolds with a 30:70 pearl/CaP ratio, demonstrating enhanced proliferation and differentiation of osteoblasts. CaP ratios and p/CaP concentrations influenced the biological response.

## Figures and Tables

**Table 1:** Details of porous chitosan scaffold composite samples prepared in this study. Pearl/CaP ratios indicate the mass ratio of pearl to CaP. Weight percentages (wt.%) are relative to chitosan.

Group of Scaffolds	Abbreviation	Particles Added to Chitosan Scaffold
Pure CS scaffolds	CS	-
Pearl/CS scaffolds	P1	10 wt.% pearl
	P3	30 wt.% pearl
	P5	50 wt.% pearl
HA/CS scaffolds	H1	10 wt.% HA
	H3	30 wt.% HA
	H5	50 wt.% HA
Pearl/CaP (1:9)(P1CaP9)/CS scaffolds	P1CaP9_10	10 wt.% P1CaP9
	P1CaP9_30	30 wt.% P1CaP9
	P1CaP9_50	50 wt.% P1CaP9
P3CaP7/CS scaffolds	P3CaP7_10	10 wt.% P3CaP7
	P3CaP7_30	30 wt.% P3CaP7
	P3CaP7_50	50 wt.% P3CaP7
P5CaP5/CS scaffolds	P5CaP5_10	10 wt.% P5CaP5
	P5CaP5_30	30 wt.% P5CaP5
	P5CaP5_50	50 wt.% P5CaP5

**Table 2:** Diameters of pores as determined from the SEM images of all scaffolds.

Names	N Total	Mean μm	Standard Deviation μm	Minimum μm	Median μm	Maximum μm	Median Absolute Deviation μm
CS	351	110.30	104.35	31.86	98.58	589.97	11.72
P1	358	121.40	115.40	32.18	99.27	595.87	22.14
P3	363	134.78	129.14	30.27	95.15	560.47	39.63
P5	373	114.50	108.88	30.19	82.98	558.97	21.52
H1	349	109.03	103.70	28.61	87.57	529.70	21.57
H3	388	118.86	113.54	28.56	91.47	528.81	27.38
H5	353	127.04	121.82	28.03	88.34	519.07	38.69
P1CaP9_10	347	121.88	116.40	29.43	83.35	544.97	28.53
P1CaP9_30	355	128.20	122.92	28.37	91.06	525.30	37.13
P1CaP9_50	358	128.93	123.07	31.49	107.73	582.67	20.23
P3CaP7_10	356	126.24	120.52	30.69	96.06	568.29	30.55
P3CaP7_30	362	138.07	132.25	31.22	107.20	578.07	20.88
P3CaP7_50	370	107.58	121.79	31.08	96.89	575.43	30.69
P5CaP5_10	349	110.85	115.96	26.24	100.49	485.95	34.36
P5CaP5_30	352	102.86	105.97	26.25	92.50	486.01	32.35
P5CaP5_50	355	99.52	104.17	28.75	88.88	532.34	27.71

**Table 3:** Densities and porosities of all sample scaffolds.

Composition	Density (g/cm^3^)	Porosity
CS	0.061	80.33 ± 10.37%
P1	0.065	81.77 ± 3.76%
P3	0.071	79.00 ± 4.49%
P5	0.076	76.23 ± 5.42%
H1	0.079	81.35 ± 3.42%
H3	0.082	79.23 ± 4.17%
H5	0.089	75.10 ± 7.27%
P1CaP9_10	0.067	78.64 ± 1.08%
P1CaP9_30	0.072	75.83 ± 5.40%
P1CaP9_50	0.075	69.25 ± 5.42%
P3CaP7_10	0.065	78.17 ± 1.18%
P3CaP7_30	0.068	76.13 ± 1.70%
P3CaP7_50	0.074	70.90 ± 6.79%
P5CaP5_10	0.064	69.73 ± 8.62%
P5CaP5_30	0.068	67.73 ± 4.62%
P5CaP5_50	0.071	63.73 ± 3.92%

## Data Availability

The data presented in this study are available on request.
